# Prevalence of mutations in the *Plasmodium falciparum* chloroquine resistance transporter, PfCRT, and association with ex vivo susceptibility to common anti-malarial drugs against African *Plasmodium falciparum* isolates

**DOI:** 10.1186/s12936-020-03281-x

**Published:** 2020-06-05

**Authors:** Francis Tsombeng Foguim, Hervé Bogreau, Mathieu Gendrot, Joel Mosnier, Isabelle Fonta, Nicolas Benoit, Rémy Amalvict, Marylin Madamet, Sharon Wein, Bruno Pradines, The French National Reference Centre for Imported Malaria Study Group, V. Augis, P. Bastien, F. Benoit-Vical, A. Berry, P. Brouqui, P. Chauvin, M. Cividin, F. Courtier, P. Delaunay, L. Delhaes, M. Drancourt, N. Dubosc, T. Gaillard, A. Genin, E. Garnotel, E. Javelle, C. L’Ollivier, J. C. Lagier, E. Ledault, M. Leveque, D. Malvy, P. Marty, G. Ménard, E. Menu, P Millet, P Minodier, P. Parola, S Picot, C. Pomares-Estran, S. Ranque, M. C. Receveur, A. Robin, E. Sappa, H. Savini, J. Sevestre, F. Simon, Y. Sterkers, C. Surcouf, E. Varlet, A. Wolff

**Affiliations:** 1 Unité Parasitologie et entomologie, Département Microbiologie et maladies infectieuses, Institut de Recherche Biomédicale des Armées, IHU Méditerranée Infection, 19-21 Boulevard Jean Moulin, 13005 Marseille, France; 2 Aix Marseille Univ, IRD, SSA, AP-HM, VITROME, Marseille, France; 3 IHU Méditerranée Infection, Marseille, France; 4 Centre National de Référence du Paludisme, Marseille, France; 5 Laboratory of Pathogen Host Interactions, UMR 5235, CNRS-Université de Montpellier, Montpellier, France

**Keywords:** Malaria, *Plasmodium falciparum*, Antimalarial drug, Resistance, In vitro, Molecular marker, *pfcrt*, I356T, Africa

## Abstract

**Background:**

The *Plasmodium falciparum* chloroquine transporter gene (*pfcrt*) is known to be involved in chloroquine and amodiaquine resistance, and more particularly the mutations on the loci 72 to 76 localized within the second exon. Additionally, new mutations (T93S, H97Y, C101F, F145I, M343L, C350R and G353V) were recently shown to be associated with in vitro reduced susceptibility to piperaquine in Asian or South American *P. falciparum* strains. However, very few data are available on the prevalence of these mutations and their effect on parasite susceptibility to anti-malarial drugs, and more particularly piperaquine in Africa.

**Methods:**

A molecular investigation of these mutations was performed in 602 African *P. falciparum* parasites collected between 2017 and 2018 on malaria patients hospitalized in France after a travel in African countries. Associations between genotypes and in vitro susceptibilities to piperaquine and standard antimalarial drugs were assessed.

**Results:**

None of the mutations, previously described as associated with piperaquine resistance, was found in the 602 *P. falciparum* African isolates. The K76T mutation is associated with resistance to chloroquine (p < 0.0002) and desethylamodiaquine (p < 0.002) in Africa. The K76T mutation is not associated with in vitro reduced susceptibility to piperaquine. The mutation I356T, identified in 54.7% (n = 326) of the African isolates, was significantly associated with reduced susceptibility to quinine (p < 0.02) and increased susceptibility to mefloquine (p < 0.04). The K76T and I356T mutations were significantly associated in West African isolates (p = 0.008).

**Conclusion:**

None of the mutations in *pfcrt* found to be associated with piperaquine reduced susceptibility in Asia or South America (T93S, H97Y, C101F, F145I, M343L C350R and G353V) were found in the 602 African isolates including the three isolates with reduced susceptibility to piperaquine. The K76T mutation, involved in resistance to chloroquine and amodiaquine, and the I356T mutation were not associated with in vitro reduced susceptibility to piperaquine. Differences in mefloquine susceptibility between I356 and 356T isolates were, while statistically different, minimal. Further analyses are needed with a more important sample size from the same geographic area to confirm the role of the I356T mutation on quinine susceptibility.

## Background

The *Plasmodium falciparum* chloroquine transporter gene (*pfcrt*) has been under different drugs pressure during parasites evolution for decades. As a result, acquisition of mutations for adaptation has emerged. These mutations induce an alteration of membrane protein physiochemical properties modifying vacuolar traffic of these drugs in resistant parasites [1]. Quinoline-based compounds like chloroquine, amodiaquine, mefloquine, primaquine, and piperaquine share a similar structure [2]. The common and widely spread mutations on *pfcrt* gene associated with resistance are localized within its second exon. Precisely, chloroquine resistance was associated with mutations on the loci 72–76 [1, 3]. The K76T mutation was associated with chloroquine resistance and used as molecular marker to survey chloroquine resistance in epidemiological studies [3]. But mutations on other loci were also found to be involved either as amplifying the resistance or as compensatory mutations for the fitness cost [4].

Since 2005, the World Health Organization (WHO) has recommended artemisinin-based combination therapy (ACT) as the first-line treatment against malaria. However, *P. falciparum* parasites resistant to artemisinin derivatives rapidly emerged in Southeast Asia, and more particularly in western Cambodia, Myanmar, Thailand and Laos [5, 6]. More recently, the emergence of *P. falciparum* resistance to dihydroartemisinin-piperaquine was observed in Cambodia, where recrudescent infections had rapidly increased [7–9], and then in Vietnam [10, 11]. Duplication of the *P. falciparum Plasmepsin 2* gene (*pfpm2*), encoding a protease involved in haemoglobin degradation, has been found to be associated with reduced in vitro susceptibility to piperaquine in Cambodian *P. falciparum* parasites and with dihydroartemisinin-piperaquine failures in Cambodia [12, 13]. However, the involvement of *pfpm2* in piperaquine resistance seems controversial in Africa [14]. Parasites from patients successfully treated with dihydroartemisinin-piperaquine could carry with two copies of *pfpm2* while only a single copy of *pfpm2* was detected in isolates collected in imported malaria cases after dihydroartemisinin-piperaquine failures or in *P. falciparum* isolates with in vitro reduced susceptibility to piperaquine [15–20]. All these data suggest that *pfpm2* would not be the only gene that explains the resistance to piperaquine in Africa. Mutations in *pfcrt* could be involved in piperaquine resistance. The K76T mutation seems to be not associated with in vitro reduced susceptibility to piperaquine [21]. However, several new mutations in *pfcrt* were found to be associated with piperaquine reduced susceptibility. Piperaquine resistance, selected by in vitro continuous piperaquine pressure on *P. falciparum* Dd2, was associated with the C101F mutation in *pfcrt* on the same exon where the loci 72–76 are located [22, 23]. In 2012 in French Guiana, dihydroartemisinin-piperaquine has not been yet officially recommended as first-line treatment against malaria but was used as self-medication by illegal gold miners in rainforest. Many years after the withdrawal of chloroquine as *P. falciparum* treatment, but still used for the treatment of *P. vivax* malaria, the prevalence of the mutant haplotype SVMNT (residues 72–76 in PfCRT) remained high (97.5%). In this context, the mutation C350R on *pfcrt* emerged in 2002 to reach a prevalence of 58% in 2012. This mutation was involved in the decrease of in vitro susceptibility to piperaquine with a restoration of chloroquine susceptibility [23]. In Cambodia, Agrawal and colleagues identified the substitution of phenylalanine by isoleucine on the locus 145 (F145I) associated with a decrease in piperaquine susceptibility [25]. In a context of dihydroartemisinin-piperaquine resistance in Cambodia and high prevalence of K13 C580Y mutation associated with artemisinin resistance, new *pfcrt* mutations (H97Y, M343L, and G353V) were revealed to induce in vitro piperaquine resistance [26, 27]. Treatment failures with dihydroartemisinin-piperaquine were associated with T93S, H97Y, F145I and I218F mutations in PfCRT and with plasmepsin 2/3 amplification in Cambodia, Thailand and Vietnam [28, 29]. Many other mutations were found in *pfcrt* but few were investigated for their association with anti-malarial drug resistance. The mutation I356T/L for instance is often found both on Asian or South-American parasites [30, 31].

These data suggest that parasite susceptibility to piperaquine is affected by some of these mutations in Southeast Asia and South America. However, very few data are available on the prevalence of these mutations and their effect on parasites susceptibility to piperaquine in Africa. It would, therefore, be essential to study these loci to provide more information on African parasites genotypes and piperaquine susceptibility. To this end, a molecular epidemiologic study of *pfcrt* genotypes was conducted on 602 African *P. falciparum* parasites collected from different countries. The association between these mutations and ex vivo susceptibility to piperaquine (PPQ) was assessed. Association with resistance to other common antimalarial drugs, such as chloroquine (CQ), quinine (QN), dihydroartemisinin (DHA), artesunate (AS) monodesethylamodiaquine (DQ), mefloquine (MQ), lumefantrine (LMF) and pyronaridine (PND) was also evaluated.

## Methods

### Sample collection

A retrospective analysis was performed on 602 African *P. falciparum* samples collected between January 2017 and October 2018 on malaria patients hospitalized in France after a travel in sub-Saharan African countries (Table 1). The samples were sent from different civilian or military hospitals of the French National Reference Centre for Imported Malaria network (Aix en Provence, Bordeaux, Lyon, Marseille, Montpellier, Nice, Toulon and Toulouse) to the French National Reference Centre for Malaria (IRBA, IHU Méditerranée Infection, Marseille, France).

**Table 1:** Number of I256T and K76T mutations per regions and countries

Regions/countries	Allele I356 or 356T (number of samples)				Allele K76 or 76T (number of samples)			
	I356	Mixed	356T	Total	K76	Mixed	76T	Total
West Africa	145	17	139	301	197	13	68	278
Benin	6	1	6	13	2	1	6	9
Burkina Faso	9	1	10	20	16	1	2	19
Gambia	1	0	0	1	0	0	1	1
Ghana	2	0	4	6	6	0	0	6
Guinea	24	5	19	48	17	5	27	49
Ivory Coast	70	6	65	141	115	3	13	131
Mali	4	0	4	8	3	1	3	7
Niger	5	0	6	11	8	0	0	8
Nigeria	6	2	8	16	6	0	7	13
Senegal	7	2	4	13	6	2	5	13
Sierra Leone	1	0	3	4	3	0	3	6
Togo	9	0	10	19	15	0	1	16
Cape Verde	1	0	0	1				
Central Africa	101	12	116	229	179	10	40	229
Angola	0	0	2	2	2	0	0	2
Cameroon	54	4	65	123	106	4	15	125
Central African Republic	14	0	20	34	32	0	0	32
Chad	5	0	11	16	10	2	2	14
Congo	11	4	8	23	8	2	13	23
Gabon	17	4	10	31	21	2	10	33
East Africa	7	0	3	10	9	0	2	11
Burundi	1	0	1	2	1	0	1	2
Djibouti	1	0	1	2	1	0	1	2
Ethiopia	1	0	0	1	1	0	0	1
Mozambique	1	0	1	2	3	0	0	3
Sudan	1	0	0	1	1	0	0	1
Tanzania	2	0	0	2	2	0	0	2
Indian Ocean	12	0	23	35	31	1	1	33
Comoros	9	0	20	29	26	0	1	27
Madagascar	3	0	3	6	5	1	0	6
Maghreb	1	1	0	2	1	0	0	1
Morocco	1	1	0	2	1	0	0	1
Unknown origin	10	2	13	25	16	2	8	26
Total	276	32	294	602	433	26	119	578

### Nucleic acid extraction

DNA extraction for each sample was performed and purified using the QIAamp^®^ DNA Mini kit according to the manufacturer’s recommendations (Qiagen, Hilden, Germany).

#### *pfcrt* genotyping

Two fragments of the *pfcrt* gene (PF3D7_0709000) were amplified. The first fragment of 840 nucleotides including the exons 2, 3 and part of exon 4 and covering the positions 72–76, 93, 97, 101, 145, 146, 158 and 159 was amplified by PCR using the primers pairs: 5′-GAT-GGC-TCA-CGT-TTA-GGT-GGA-3′ and 5′-TGT-TAC-AAC-AAT-AAT-AAC-TGC-TCC-G-3′. The second fragment of 339 nucleotides including the exon 10 and covering the positions 343, 350, 353 and 356 was amplified using the primer pairs: 5′-CCA-TAT-AAT-TTT-TCA-TTT-TC-3′ and 5′-GTT-CTC-TTA-CAA-CAT-CAC-3′. The reaction mixture for PCR contained 200 ng of genomic DNA, 0.32 µM of each primer, 1X final of reaction buffer (750 mM Tris-HCl, 200 mM (NH4)_2_SO4, 0.1% (v/v) Tween 20 and stabilizer, pH 8.8), 2.5 mM MgCl2, 200 µM of dNTP mixture (Euromedex, Souffelweyersheim, France) and 0.2 U of Hot Diamond Taq^®^ polymerase (Eurogentec, Liège, Belgium) in a final volume of 25 µL. For the first fragment, the thermal cycler (Life Eco V 2.04; Bioer, China) was programmed as follows: 95 °C for 5 min, 40 cycles of 95 °C for 30 s, 65 °C of hybridization temperature for 1 min, elongation at 65 °C for 1 min 30 s, and a final 10-min extension step at 65 °C. The amplification programme for the second fragment was 95 °C for 5 min, 40 cycles of 95 °C for 30 s, hybridization temperature 46 °C for 45 s, elongation at 68°C for 1 min, and a final 10 min extension step at 68 °C. To cover the mutations T93S, H97Y and C101F, the purified amplicons were sequenced by the amplification forward primer of the first fragment and the mutations F145I, I146L, F158L and V159S were sequenced using the primer 5′-TTA-GGA-ACG-ACA-CCG-AAG-C-3′. To cover the mutations M343L, C350R and I356T, the amplified second fragment was purified and sequenced using the forward primer. Sequencing was performed on ABI Prism 3100 analyser (Applied Biosystems, Villebon sur Yvette, France) according to the manufacturers’ instructions. Base calling was implemented on Vector NTI 10.3.0 software (Invitrogen, Cergy Pontoise, France). Poor-quality sequences were either re-sequenced or discarded and repeat polymorphisms were retained for analysis if clean individual peaks were observed in the electropherogram.

### Drugs and ex vivo assay

The drug susceptibility assays were performed using the HRP2 ELISA-based assay Malaria Ag Celisa kit (ref KM2159, Cellabs PTY LDT, Brookvale, Australia) as previously described [32].

Each batch of plates was validated using the CQ-resistant W2 strain (isolated from Indochina; MRA-157 obtained from MR4, VA, USA) in four independent experiments. The mean 50% inhibitory concentration (IC_50_) values for all the batches used over 2 years were 495 ± 45 nM for CQ, 401 ± 36 nM for QN, 93 ± 17 nM for DQ, 23.9 ± 3.4 nM for MQ, 57.1 ± 5.6 nM for PPQ, 18.6 ± 3.1 nM for PND, 2.4 ± 0.4 nM for DHA and 1.6 ± 0.4 nM for AS. A comparison of the W2 anti-malarial susceptibility data between the different batches of plates indicated that there was no significant difference in the responses to anti-malarial drugs over the 2 years (0.625 < p < 0.990).

The polymorphic genetic markers *msp1* and *msp2* and microsatellite markers specific to *P. falciparum* W2 were genotyped at least once a month to verify W2 clonality as previously described [33–35].

### Data management and statistical analysis

IC_50_ values were calculated with the inhibitory sigmoid E_max_ model, with estimation of the IC_50_ through non-linear regression using a standard function of the R software ICEstimator version 1.2 (http://www.antimalarial-icestimator.net/). IC_50_s estimates values and sequences analysis were collected and analyzed on excel sheets and GraphPad Prism (V7.0a). Samples were grouped by regions for analysis to reflect parasites population structure sharing the same genetic background. The median of the IC_50_ values was calculated for parasites with identical genotypes. Comparison between groups was implemented with Mann-Whitney test. Proportions were compared by Chi square test or Fisher exact test depending on sample size at significance level of 0.05.

## Results

### *pfcrt* mutations

No mutations were detected on the loci 93, 97, 101, 145, 146, 158, 159, 343 and 350 among the 602 *P. falciparum* samples. Polymorphism on the locus 356 (I356T) was found in 54.7% (n = 326) of samples (mutants and mixed infection). More precisely, 276 isolates (45.8%) carried the wild type (I356) allele, 294 isolates (48.8%) carried the mutant allele (356T) and 32 isolates (5.4%) were mixed with both wild type and mutant allele (Table 1). Among the 578 samples tested for the 72–76 haplotype (24 were not genotyped successfully), the overall proportion of wild allele K76 was 74.9% (n = 433) versus 20.6% (n = 119) for mutant allele 76T and 4.5% (n = 26) for mixed populations (K76 and 76T) (Table 1). Details of I356T and K76T mutations per country are presented in Table 1. The majority of samples were from West African followed by Central African countries. Because other regions had low samples size, only West African and Central African samples were considered for further analysis. No association between K76T and I356T mutations was found for Central African isolates (n = 209, p = 0.1) while for West African isolates, there was a significant association found (n = 248, p = 0.008).

### Drug susceptibility

**Fig. 1:**
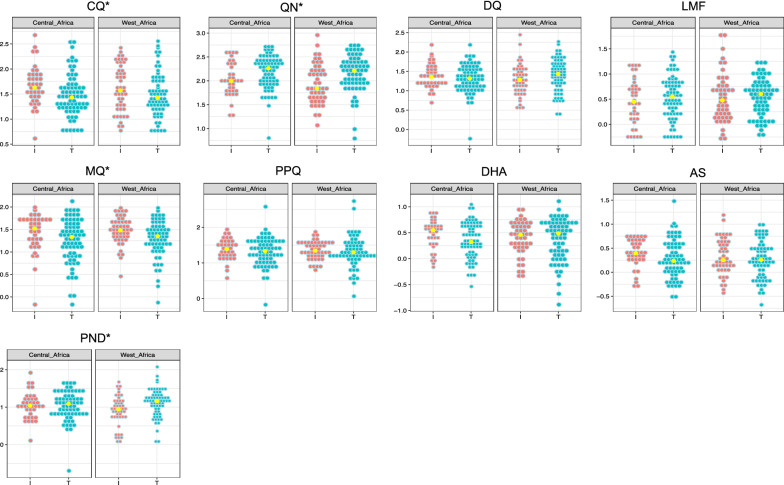
Dot plot of the IC_50_ values distribution of *P. falciparum* isolates from West Africa and Central Africa for chloroquine (CQ), quinine (QN), desethylamodiaquine (DQ), lumefantrine (LMF), mefloquine (MQ), piperaquine (PPQ), dihydroartemisinin (DHA), artesunate (AS) and pyronaridine (PND) according to I356T. Each dot represents the log10(IC_50_) for each isolate, red dots represent wild type I356 isolates (I), blue dots mutant 356T isolates (T) and yellow dots the log10(median IC_50_) value for each drug. The y-axis represents the log10(IC_50_) in nM. * Significant differences were found between wild type (I356) and mutant (356T) in West Africa (QN: p = 0.001, MQ: p = 0.01, PND: p = 0.005) and in Central Africa (QN: p = 0.02, MQ: p = 0.04, CQ: p = 0.01)

On the 602 samples collected, 296 isolates (117 from West Africa and 113 from Central Africa) were successfully evaluated in vitro. Values of IC_50_ estimates ranged from 0.7 nM to 544.0 nM for PPQ (mean = 27.9 nM), 4.1 nM to 474.2 nM for CQ (mean = 59.6 nM), 6.2 nM to 906.3 nM for QN (mean = 179.6 nM), 0.5 nM to 61.5 nM for LMF (mean = 5.2 nM), 2.5 nM to 185.1 nM for DQ (mean = 31.2 nM), 0.6 nM to 133.5 nM for MQ (mean = 30.5 nM), 0.2 nM to 119.3 nM for PND (mean = 16.4 nM), 0.2 nM to 18.5 nM for DHA (mean = 3.8 nM) and 0.2 nM to 64.0 nM for AS (mean = 2.6 nM) (Fig. 1). Based on the cut-off values for reduced in vitro susceptibility to CQ (100 nM), QN (800 nM), DQ (80 nM), MQ (30 nM), LMF (150 nM), PPQ (135 nM), (PND 60 nM), DHA and AS (10.5 nM) [36, 37], a proportion of samples had an in vitro reduced susceptibility to CQ (16.3%) and MQ (40.2%).

IC_50_ values were classified in 2 groups based on their genotype for the locus 356. The average parameters estimates of IC_50_ values for wild type (I356) and mutant allele (356T) for samples from West African and Central African countries are given in Table 2. There was a significant difference between the alleles I356 or 356T and IC_50_ median values of QN (p = 0.001) and MQ (p = 0.01) in West African group and QN (p = 0.02), CQ (p = 0.01), and MQ (p = 0.04) in Central Africa group (Table 2).

**Table 2:** IC_50_ average parameters in nM for wild type (I356) and mutant (356T) *P. falciparum* isolates from West Africa and Central Africa for piperaquine (PPQ), quinine (QN), mefloquine (MQ), chloroquine (CQ), lumefantrine (LMF), desethylamodiaquine (DQ), pyronaridine (PND), dihydroartemisinin (DHA) and artesunate (AS)

Drugs	West Africa					Central Africa				
	Wild type I356		Mutant 356T		p value	Wild type I356		Mutant 356T		p value
	Median (no)	Min-Max	Median (no)	Min-Max		Median (no)	Min–Max	Median (no)	Min–Max	
PPQ	22.2 (49)	6.8–76.3	19.9 (66)	1.2–544	0.32	24.5 (36)	4.1–79	21.4 (70)	0.67–96.3	0.4
QN	69.1 (49)	11.7–906.3	162.6 (68)	6.2–590.5	*0.001*	98.2 (36)	17.6–365.2	183.4 (73)	28.9–538.4	*0.02*
MQ	30.4 (49)	2.9–89.8	22.4 (67)	0.8–100.6	*0.01*	33.3 (38)	0.7–97.9	21.5 (72)	0.62–133.9	*0.04*
CQ	37.4 (49)	6.2–283.2	26.5 (68)	5.5–359.5	0.1	42.8 (39)	4.1–474.2	26.9 (73)	5.79–325	*0.01*
LMF	3.0 (49)	0.5–61.54	4.0 (68)	0.6–18.2	0.6	2.8 (40)	0.6–15.5	3.6 (73)	0.55–22.4	0.5
DQ	19.2 (49)	3.4–169.2	27.1 (68)	2.5–185.1	0.07	22.8 (40)	5.1–152.8	20.8 (73)	4.7–152.9	0.4
PND	8.8 (48)	1.2–50.1	14.5 (66)	0.7–119.3	*0.005*	11.1 (38)	3.9–41	12.3 (67)	0.2–47.9	0.8
DHA	2.9 (49)	0.4–8.3	3.1 (68)	0.2–12.8	0.9	3.5 (40)	0.7–7.7	2.1 (73)	0.47–11.1	0.08
AS	1.8 (49)	0.4–15.48	1.8 (67)	0.2–9.8	0.6	2.5 (39	0.6–5.7	1.7 (71)	0.29–30.2	0.05

West Africa: Benin, Burkina Faso, the Gambia, Ghana, Guinea, Ivory Coast, Niger, Mali, Nigeria and Togo

Central Africa: Cameroon, Central African Republic, Chad, Gabon and Republic of Congo

Italic p values refers to significant difference (p < 0.05)

Parasites harbouring the K76T mutation were significantly less susceptible to CQ (p < 0.0002) and DQ (p < 0.002) in Africa (Table 3). PPQ susceptibility was not significantly associated with the K76T mutation.

**Table 3:** IC_50_ average parameters in nM for wild type (K76) and mutant (76T) *P. falciparum* isolates from West Africa and Central Africa for piperaquine (PPQ), quinine (QN), mefloquine (MQ), chloroquine (CQ), lumefantrine (LMF), desethylamodiaquine (DQ), pyronaridine (PND), dihydroartemisinin (DHA) and artesunate (AS)

Drugs	West Africa					Central Africa				
	Wild type K76 (n = 76)		Mutant 76T (n = 27)		p value	Wild type K76 (n = 82)		Mutant 76T (n = 14)		p value
	Median	Min–Max	Median	Min–Max		Median	Min–Max	Median	Min–Max	
CQ	23.2	5.5–229.6	152.6	7.9–381	*4.295E−10*	28.7	5.1–474.4	113.1	14.2–325	*0.0002*
DQ	19.3	2.5–185.1	46.2	6.5–169.2	*0.0002*	20.1	4.7–85.8	32.9	16.3–152.9	*0.002*
QN	138.6	6.2–906.3	163.8	36.7–586	0.2	143.9	28.9–538.4	180.1	17.6–503	0.52
LMF	3.9	0.5–31.6	3.5	0.5–61.5	0.3	3.1	0.5–27.3	4.9	0.6–14.2	0.758
MQ	25.2	0.8–89.8	26.2	2.9–76.7	0.8	23.7	1.04–86.6	40.9	0.7–92.3	0.11
DHA	2.9	0.2–12.8	2.8	0.5–8.2	0.9	2.9	0.5–18.5	2.9	0.7–6.5	0.78
PND	11.8	1.1–119.3	13.3	1.9–50.2	0.7	11.3	0.2–46.6	12.3	2.7–40.8	0.99
AS	1.8	0.3–64	2.1	0.4–7.3	0.8	1.9	0.3–30.2	3.0	0.6–5.9	0.36
PPQ	20.2	1.2–78.9	28.6	7.4–544	0.08	20.6	0.7–84.6	20.9	3.4–67.1	0.82

West Africa: Benin, Burkina Faso, Gambia, Ghana, Guinea, Ivory Coast, Niger, Mali, Nigeria and Togo

Central Africa: Cameroon, Central African Republic, Chad, Gabon and Republic of Congo

Italic p values refers to significant difference (p < 0.05)

## Discussion

Understanding the genetic profile of drug resistance genes in *P. falciparum* malaria in endemic countries is essential. Knowing the predominant genotype circulating in a country or a region provide to policy makers a valuable information for the treatment regiments to be adopted depending on the predominant resistance genetic marker.

Mutations within the *pfcrt* haplotype 72–76 are known to confer resistance to chloroquine and other quinoline drugs, like amodiaquine and lumefantrine [1, 3, 38, 39]. As already documented many dozens of times, the present results show that the K76T mutation is associated with in vitro resistance to chloroquine and desethylamodiaquine and is an excellent molecular marker of resistance to chloroquine and amodiaquine for resistance monitoring in Africa. In contrast, the K76T mutation did not appear to be associated with in vitro reduced susceptibility to piperaquine (Table 3), confirming previous results [21, 38].

Previous studies found several new mutations in *pfcrt* associated with piperaquine reduced susceptibility, like the mutations T93S, H97Y, C101F, F145I, M343L and G353V in Cambodian parasites [22, 23, 25–29] and C350R in isolates from French Guiana [24] and Suriname [30]. However in studies on African *P. falciparum* samples, there was no report of identification of these mutations [39, 40]. None of these mutations was identified in the present 602 African isolates. These data suggest a very low prevalence of these mutations in African parasites, certainly due to the very low prevalence of *P. falciparum* parasites resistant to piperaquine in Africa. The present results suggest that susceptible parasites to piperaquine do not carry these mutations before selection pressure. The three isolates with piperaquine IC_50_ above the threshold of reduced susceptibility (135 nM) carried none of these mutations. Moreover, the absence of mutation on codon 93, 97, 101, 145, 146, 158, 159, 343 and 350 suggest that these mutations are not associated with in vitro resistance to amino-quinoline (chloroquine, amodiaquine) or amino-alcohol compounds (mefloquine) in Africa.

The mutation I356T found in 54.7% of isolates in the present work was already identified. The I356T mutation was previously reported at a rate of 24% in *P. falciparum* parasites from Malaysia [41]. In a local and global epidemiological study on global spread of mutant PfCRT, the I356T mutation was found in 12.6% of the *P. falciparum* isolates tested in Africa in 2011–2012 and mainly in the Gambia (78.7%) and the Democratic Republic of Congo (36.5%), and in 70.1% of isolates from Asia and from Thailand (99.2%) and Cambodia (67.7%) [42]. The I356T mutation was detected in 2.4% of isolates, as mixed infections (I356 and 356T alleles) collected before artemether-lumefantrine treatment in Uganda in 2014 [43]. This mutation was not found in *P. falciparum* positively isolates after artemether-lumefantrine treatment. A different amino acid substitution on the locus 356 (I356L) was also found in isolates from Latin America [1, 30], but this mutation was absent the present 602 African isolates. However, this mutation was not observed in isolates from Suriname [30].

The present study shows that the mutation I356T seems to be involved in quinine and mefloquine susceptibilities in African *P. falciparum* parasites. The mutation I356T is significantly associated with reduced susceptibility to quinine and increased susceptibility to mefloquine (Table 2). This mutation is also significantly associated with increased susceptibility to chloroquine but only in isolates from Central Africa. Additionally, this mutation is significantly associated with decreased susceptibility to pyronaridine but only in West African parasites. This mutation is not associated with piperaquine susceptibility, arguing against a role for residue 356 in modeling *P. falciparum* susceptibility to piperaquine. These data confirm the results of Dhingra et al. [42], that showed no difference in the survival of *P. falciparum* parasites with the FCB (I356) and Dd2 (356T) alleles in the presence of piperaquine. Additionally, the I356T mutation was not associated with ex vivo susceptibility to lumefantrine in the present 602 isolates according to previous data showing that this mutation was not selected in recrudescent *P. falciparum* parasites after artemether-lumefantrine [43].

Allelic frequencies of K76T and I356T in Central Africa show that there is no association with the two alleles. But on West African isolates, a significant association (p = 0.008) was found.

None of the mutations in PfCRT associated with in vivo piperaquine resistance in Asia or in vitro resistance in South America (T93S, H97Y, C101F, F145I, M343L, C350R and G353V) were found in the three isolates with ex vivo reduced susceptibility to piperaquine. All these isolates carried the I356T mutation and two the K76T mutation. Only one copy of *pfpm2* was detected in the three isolates in a previous work [19]. But one of the weaknesses of the study is the low number of samples with reduced susceptibility to piperaquine. Further investigations are required to understand piperaquine resistance in Africa where dihydroartemisinin-piperaquine treatment remains highly effective [44].

## Conclusion

None of the mutations in *pfcrt* found to be associated with piperaquine reduced susceptibility in Asia or South America (T93S, H97Y, C101F, F145I, M343L C350R and G353V) were found in the 602 African isolates including the three isolates with reduced susceptibility to piperaquine. The K76T mutation, involved in resistance to chloroquine and amodiaquine, and the 356T mutation were not associated with in vitro reduced susceptibility to piperaquine. Differences in mefloquine susceptibility between I356 and 356T isolates were, while statistically different, minimal. Further analyses are needed with a more important sample size from the same geographic area to confirm the role of the I356T mutation on quinine susceptibility.

## Data Availability

The datasets analysed in this study are available from the corresponding author on reasonable request.

## Abbreviations

AS: Artesunate
CQ: Chloroquine
DHA: Dihydroartemisinin
DNA: Deoxyribonucleic acid
dNTP: Deoxynucleotide triphosphate
DQ: Monodesethylamodiaquine
ELISA: Enzyme linked immunosorbent assay
HRP2: Histidine-rich protein 2
IC_50_: 50% inhibitory concentration
LMF: Lumefantrine
MQ: Mefloquine
PCR: Polymerase chain reaction
PfCRT: *Plasmodium falciparum* chloroquine resistance transporter
PfK13: *Plasmodium falciparum* Kelch 13
*Pfpm2*: *Plasmodium falciparum* plasmepsin 2 gene
PND: Pyronaridine
PPQ: Piperaquine
QN: Quinine
WHO: World Health Organization
